# Genome editing in East African cichlids and tilapias: state-of-the-art and future directions

**DOI:** 10.1098/rsob.230257

**Published:** 2023-11-29

**Authors:** Bethan Clark, Muktai Kuwalekar, Bettina Fischer, Joost Woltering, Jakob Biran, Scott Juntti, Claudius F. Kratochwil, M. Emília Santos, Miguel Vasconcelos Almeida

**Affiliations:** ^1^ Department of Zoology, University of Cambridge, Cambridge, UK; ^2^ Department of Genetics, University of Cambridge, Cambridge, UK; ^3^ Department of Biochemistry, University of Cambridge, Cambridge, UK; ^4^ Wellcome/CRUK Gurdon Institute, University of Cambridge, Cambridge, UK; ^5^ Helsinki Institute of Life Science (HiLIFE), University of Helsinki, Helsinki, Uusimaa 00014, Finland; ^6^ Faculty of Biological and Environmental Sciences, University of Helsinki, Helsinki, Uusimaa 00014, Finland; ^7^ Zoology and Evolutionary Biology, Department of Biology, University of Konstanz, Konstanz, Baden-Württemberg 78457, Germany; ^8^ Department of Poultry and Aquaculture, Institute of Animal Sciences, Agricultural Research Organization, Volcani Center, Rishon Lezion, Israel; ^9^ Department of Biology, University of Maryland, College Park, MD, USA

**Keywords:** CRISPR/Cas9, genome editing, tilapia, emerging model organisms, East African cichlids

## Abstract

African cichlid fishes of the Cichlidae family are a group of teleosts important for aquaculture and research. A thriving research community is particularly interested in the cichlid radiations of the East African Great Lakes. One key goal is to pinpoint genetic variation underlying phenotypic diversification, but the lack of genetic tools has precluded thorough dissection of the genetic basis of relevant traits in cichlids. Genome editing technologies are well established in teleost models like zebrafish and medaka. However, this is not the case for emerging model organisms, such as East African cichlids, where these technologies remain inaccessible to most laboratories, due in part to limited exchange of knowledge and expertise. The Cichlid Science 2022 meeting (Cambridge, UK) hosted for the first time a Genome Editing Workshop, where the community discussed recent advances in genome editing, with an emphasis on CRISPR/Cas9 technologies. Based on the workshop findings and discussions, in this review we define the state-of-the-art of cichlid genome editing, share resources and protocols, and propose new possible avenues to further expand the cichlid genome editing toolkit.

## Graphical abstract

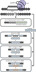

## 1. Introduction

With 26 000 species described and estimated to comprise 96% of all living fish, teleosts are the most diverse group of Actinopterygii, the ray-finned fishes [1]. Cichlid fishes (Cichlidae family), in turn, comprise a highly diverse and species-rich family of teleost fishes [2,3]. Cichlids dwell in lacustrine and riverine environments across South and Central America, Africa and India. The Cichlidae family has evolved in a particularly explosive fashion in the East African Great Lakes (Lakes Victoria, Tanganyika and Malawi/Nyasa) and surrounding bodies of water. In fact, more than 1200 cichlid species are predicted to have evolved in East Africa in the last 10 million years, representing one of the most paradigmatic examples of adaptive radiation in vertebrates [2,4–6]. Their taxonomic diversity is matched with great phenotypic, ecological and behavioural diversity. Tilapias comprise a species assemblage of African cichlids related to the cichlid radiations of the East African Great Lakes. Tilapia species have a broad natural distribution across the African continent and great economic importance, as they represent the second most produced group of fish in aquaculture worldwide [7].

Most cichlid species can be grown in aquaria and in a controlled laboratory setting. Their astonishing evolutionary history and diversity make them, collectively, enticing models to study a variety of important biological aspects, including evolution and development, sensory biology and sex determination [3]. Notably, attempts to understand cichlid evolution through genomics have become particularly popular [2,3,5,8,9]. This is demonstrated by the eight cichlid genomes currently available at Ensembl (Release 109, February 2023), seven of which are from African cichlids. Furthermore, a large community effort is constantly expanding the amount of available genomic data.

Given their diversity and the availability of genomic resources, cichlids are often employed in genotype–phenotype association studies and speciation research. To clearly identify direct links between the genotype and the phenotype, functional studies should be employed [10]. However, these are still largely lacking in cichlids due to the challenges of establishing functional genomic tools, a problem shared with other emerging model organisms [10]. Transposase-mediated transgenesis, transcription activator-like effector nucleases (TALEN) and clustered regularly interspaced short palindromic repeats (CRISPR)/Cas9 genome editing technologies have been implemented in tilapia and other cichlids (table 1), mostly to knock out genes involved in pigmentation, reproduction, and behaviour. However, because of a limited exchange of knowledge and expertise, these techniques remain largely inaccessible to most laboratories working with cichlids. To address this, the Cichlid Science 2022 meeting, which took place in September 2022 in Cambridge, UK, hosted a Genome Editing Workshop for the first time. The cichlid research community met and discussed recent advances in CRISPR/Cas9 technology in cichlids; these discussions reflected the need for a synthesis and have inspired this review of the state-of-the-art of cichlid genome editing, with a particular focus on CRISPR/Cas9-mediated genome editing.

**Table 1.** Published genome edits in African cichlids.

function	refs	gene(s) targeted	method	species	phenotype
behaviour	[11]	*ptgfr*	CRISPR NHEJ	*Astatotilapia burtoni*	females with defective sexual behaviour/hormonal signalling
cell line system establishment	[12]	multiple target genes tested (including *impa1.1, nfat5*, and *nr3c1*)	CRISPR NHEJ	*Oreochromis mossambicus* cell line	N/A
conditional expression	[13]	*tiHsp70::SB11*	SB transgenesis	*Oreochromis niloticus*	induced expression of SB11 gene after heat shock and introduction of Cre/loxP system
development and control of cell fate	[14]	*gr1b*	CRISPR NHEJ	*Astatotilapia burtoni*	homeotic transformation of spines and soft rays
	[15]	*wt1a/b*	CRISPR NHEJ	*Oreochromis niloticus*	(for *wt1a*) larval lethal, required for kidney development
	[16]	*mlc3::RFP*	Tol2 transgenesis	*Archocentrus nigrofasciatus*	cell-type fluorescent labelling, developmental patterns of muscle marker promoter
gene and transposable element silencing and germline development	[17]	*piwil2*	CRISPR NHEJ	*Oreochromis niloticus*	fewer PGCs than wild-type
	[18]	*dnmt3aa/ab*	CRISPR NHEJ	*Oreochromis niloticus*	(*dnmt3aa*) gametogenesis defects
immune response	[19]	*mylz2::th2–3*	Tol2 transgenesis	*Archocentrus nigrofasciatus*	over-expression; inhibited bacterial loading for one of two bacterial species infections
pigmentation	[20]	*oca2* and 3' UTR of *oca2*	CRISPR NHEJ and HDR	*Astatotilapia calliptera*	amelanism
	[21]	*agrp2*	CRISPR NHEJ	*Pundamilia nyererei*	reappearance of horizontal stripe
	[22]	*tyr1*	CRISPR NHEJ	*Astatotilapia burtoni*	amelanism
	[23]	*pmel17*	CRISPR NHEJ	*Oreochromis mossambicus*	golden coloration in black tilapias
	[24]	*csf1ra*	CRISPR NHEJ	*Oreochromis niloticus*	two colour patterns: grey body and grey body with black tail
	[25]	*tyrb*	CRISPR NHEJ	*Oreochromis niloticus*	amelanism
	[26]	*slc45a2*	CRISPR NHEJ	*Oreochromis niloticus*	amelanism
	[27]	*hsp4*	CRISPR NHEJ	*Oreochromis niloticus*	amelanism
	[28]	*pmela/b*	CRISPR NHEJ	*Oreochromis niloticus*	reduction of melanophore number
	[29]	total of 25 genes (including *bco2b*, *csf1ra*, *gata2*, *gch2*, *hsp4*, *kita*, *kitlga*, *mitfa/b*, *pax7b*, *tyra/b*) involved in several pigmentation pathways	CRISPR NHEJ	*Oreochromis niloticus*	variable
reproduction and sex determination	[30,31]	*ARɑ/β*	CRISPR NHEJ	*Astatotilapia burtoni*	modulation testes mass and abnormal sex ratio
	[32]	*eEF1A1b*	CRISPR NHEJ	*Oreochromis niloticus*	spermatogenesis arrest, male infertility
	[33]	*foxl3* and *dmrt1*	CRISPR NHEJ	*Oreochromis niloticus*	germ cell sex-reversal
	[34]	*pgr*	CRISPR NHEJ	*Oreochromis niloticus*	subfertility
	[35]	*aldh1a2* and *cyp26a1*	CRISPR NHEJ	*Oreochromis niloticus*	shifts in timing of meiotic initiation
	[36]	*FSH::GFP*	Tol2 transgenesis	*Oreochromis niloticus*	cell-type fluorescent labelling; reproductive markers higher in dominant males
	[37,38]	*gsdf*	CRISPR NHEJ	*Oreochromis niloticus*	female infertility
	[39]	*tsp1a*	CRISPR NHEJ	*Oreochromis niloticus*	more oogonia, fewer secondary growth follicles
	[40]	*dmrt1*, *foxl2*, *cyp19a1a*	TALEN	*Oreochromis niloticus*	various sex-reversal effects
	[41]	*nanos2/3*, *dmrt1*, and *foxl2*	CRISPR NHEJ	*Oreochromis niloticus*	for *nanos2/3* loss of germ cells and female-to-male sex reversal
	[42]	*amhy*	CRISPR NHEJ	*Oreochromis niloticus*	male to female sex reversal
	[43]	*igf3*	CRISPR NHEJ	*Oreochromis niloticus*	sperm developmental defects, subfertility
	[44]	*stAR2*	CRISPR NHEJ	*Oreochromis niloticus*	meiotic delay in testes
	[45]	several microRNAs and the 3' UTR of *vasa*	CRISPR NHEJ and HDR	*Oreochromis niloticus*	decrease of *vasa* expression upon depletion of its 3' UTR
	[46]	*amhr2*	CRISPR NHEJ	*Oreochromis niloticus*	excessive germ cell proliferation
	[47]	*gnrh1:egfp*	Tol2 transgenesis	*Astatotilapia burtoni*	regulation of hormone synthesis
	[48]	*foxh1*	CRISPR NHEJ	*Oreochromis niloticus*	oogenesis arrest and infertility
	[49]	*sox30*	CRISPR NHEJ	*Oreochromis niloticus*	sperm defects and male subfertility
	[50]	*β-cat1*, *β-cat2*	TALEN	*Oreochromis niloticus*	delayed ovarian differentiation and upregulated masculinizing genes
	[51]	*sf-1*	CRISPR NHEJ	*Oreochromis niloticus*	sex reversal, gonadal dysgenesis
	[52]	*esr1*, *esr2a/b*	CRISPR NHEJ	*Oreochromis niloticus*	(for *esr2a/b*) gamete and gonad developmental defects and infertility
	[53]	*dmrt6*	CRISPR NHEJ	*Oreochromis niloticus*	fewer spermatocytes, lower ketotestosterone
	[54]	*Cyp19a1a* and *foxl2*	CRISPR NHEJ	*Oreochromis niloticus*	female-to-male sex reversal
	[55]	*Cyp19a1b*	CRISPR NHEJ	*Oreochromis niloticus*	testicular atrophy and efferent duct fibrosis
ubiquitous fluorescence	[56]	*EF1α::GFP*	Tol2 transgenesis	*Oreochromis niloticus*	ubiquitous GFP labelling
	[57]	*EF1α::GFP*	Tol2 transgenesis	*Astatotilapia burtoni*	ubiquitous GFP labelling
	[58]	*ubiquitin::eGFP*, *ubiquitin::tdTomato*, *mitfa::eGFP*	Tol2 transgenesis	*Amphilophus citrinellus*	ubiquitous GFP labelling (*ubiquitin::eGFP*) and transient labelling (*ubiquitin::tdTomato*, *mitfa::eGFP*)
vision	[59]	*mitfa*	CRISPR NHEJ	*Astatotilapia burtoni*	altered expression of *sws2a*

In bacteria and archaea, a broad repertoire of CRISPR/Cas systems serve as an adaptive immune system against viruses and plasmids [60,61]. The Cas9 protein of a type II CRISPR/Cas system of *Streptococcus pyogenes* was initially found to act as a dual RNA-guided endonuclease [62]. Since this discovery, Cas9 has become an extensively used genome editing tool, an integral part of the molecular biology and genetics experimental toolbox.

**Figure 1.**
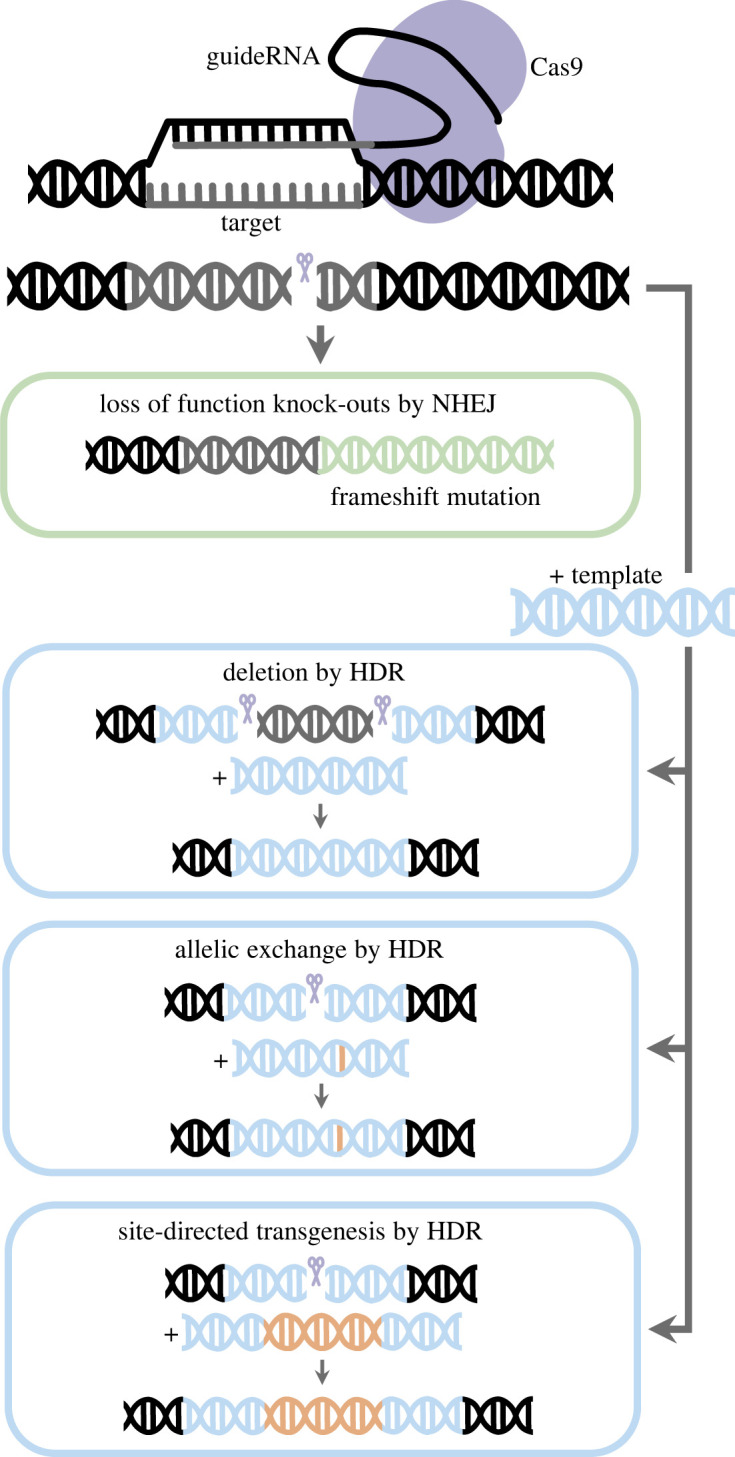
Example uses of repair mechanisms following CRISPR/Cas9 cleavage. Repair by NHEJ can cause small indels leading to frameshift mutations (green) but note that mutations may be in-frame. Repair by HDR, using a template with homology arms (blue), can be used to generate large deletions, or knock-ins, such as allelic exchange and site-directed transgenesis (orange). Scissor symbols indicate Cas9 cut site.

Cas9 requires two RNAs to find and cleave its targets: a CRISPR RNA (crRNA) and a trans-activating crRNA (tracrRNA) [62]. The former is required to recognize a target with sequence complementarity, whereas the latter serves as a binding scaffold for the Cas9 nuclease. Target specificity is achieved with a 5'NGG protospacer adjacent motif (PAM), adjacent to the target sequence complementary to the crRNA. Without a PAM in the target DNA, Cas9 will not cleave target DNA. Importantly, a single RNA chimaera of the crRNA and tracrRNA, or single guide RNA (sgRNA), also achieves target recognition and cleavage. In the context of genome editing, Cas9 can be introduced or expressed in a cell with either crRNA/tracrRNA or sgRNA complementary to a specific genomic target sequence adjacent to a 5'NGG PAM. Genome editing occurs after Cas9 cleavage, when the double-stranded DNA break (DSB) is repaired by endogenous DNA repair pathways [63,64] (figure 1). Here, we will focus on two major pathways resolving DSBs: non-homologous end joining (NHEJ) and homology-directed repair (HDR). In the error-prone NHEJ repair pathway, small insertion or deletion (indel) mutations tend to be introduced upon the re-ligation of broken DNA ends [63,64] (figure 1). Conversely, in HDR homologous recombination resolves the DSB in a templated manner. Therefore, to promote HDR, a donor template with sequences homologous to the regions flanking the Cas9 cut site(s) must be introduced into the cell, along with Cas9 and its guide RNA(s). HDR can produce precise deletions, knock-in additional sequences or allelic exchanges (figure 1). Several types of HDR pathways exist [63], but for simplicity we will not differentiate.

CRISPR/Cas9 genome editing has now been employed in a variety of animal model systems and fish are no exception: medaka and zebrafish have well established protocols [65–69]. However, this is not the case for other emerging fish models. In this review, we synthesize the state-of-the-art of CRISPR/Cas9-mediated genome editing in East African cichlids and tilapias, and propose areas of future development. This review is intended to provide guidelines, to call attention to pitfalls, and to serve as a consultation resource for cichlid and other non-model teleost laboratories embarking on CRISPR/Cas9 implementation.

## 2. State-of-the-art of CRISPR/Cas9-based genome editing in cichlids

### 2.1. Strategy

Genome editing in cichlid fishes, like other non-model teleost organisms, still presents a significant investment of time and resources. Therefore, the key initial consideration is which genome editing strategy is best to tackle your biological question, giving a clear phenotypic and/or molecular outcome and rewarding the effort invested.

The primary consideration for strategy design is recognizing what outcome(s) would be informative for your biological question, as this determines which types of sequence edits are required. What outcome would be informative depends on the type of data from which candidate genes were identified (e.g. phenotype–genotype associations, expression patterns). For example, a loss-of-function is an informative outcome when the aim is to ascertain the role of the locus in wild-type (WT) development or physiology, whereas an altered allele may provide an informative outcome when elucidating the functional relevance of a SNP. To illustrate, when the locus of interest was identified using phenotype–genotype associations such as pedigree-based mapping for quantitative trait loci (QTL) or genome wide association study (GWAS) analyses, in most cases the genetic variation identified does not correspond to a loss-of-function allele, and thus its phenotypic effect is unlikely to be mimicked by a full knock-out of the gene. Rather, genetic variation in the focal locus will correspond to a more subtle coding or regulatory variant and therefore an experimentally induced loss-of-function mutation might fail to inform on its relevance in context of the studied phenotype. In these cases, a more subtle knock-in gene editing approach would be required, which is more complicated to achieve, requires sufficient understanding of the genetic variant, and increases experimental efforts. In cases of QTLs, it is important to consider effect size, whereby a lack of discernible phenotypes is more likely to be the case if the candidate gene is of small effect. It is therefore helpful to ask whether the likely outcome of a null allele would be informative at all, or alternatively, whether it is possible to attempt the swap of a regulatory sequence (enhancer, suppressor, methylation site etc.) or coding sequence.

Where the informative outcome is a loss-of-function, knocking-out loci is most readily achieved with NHEJ to induce frameshift mutations, or alternatively using HDR for precise deletions. There are two features to take into consideration when planning to knock-out your candidate gene. First, teleost genomes have undergone an extra round of whole-genome duplication compared to other vertebrates [70]. As a consequence, many genes have gene duplicates, or paralogues, which may have redundant or partially redundant functions. Upon knock-out of your target gene, no discernible—or a rather subtle—mutant phenotype may be observed when a trait is specified together with a non-targeted paralogue. Such compensatory function may also be achieved by genetic compensation, whereby upon knock-out of a gene, a non-mutated paralogue assumes a compensatory role by an increase in transcription [71]. In these scenarios, mutant phenotypes will only become apparent if additional gene(s) are mutated. In addition to screening for paralogues within your chosen species' genome, a common approach to assess the likelihood of the outcome of a null allele is to consult published work on homologues of your gene of interest in closely related species. If your gene of interest is evolutionarily conserved across teleosts, ZFIN (zfin.org), the zebrafish database, contains a wealth of phenotypic data. Where paralogues exist, targeting multiple loci can be part of the gene editing strategy. This can be via first producing the individual mutant lines separately and subsequently crossing the different mutated alleles together, or alternatively, through combining sgRNAs targeting the different paralogues in the injection mix and later sorting out the different mutant alleles by genotyping. Of course, both strategies are accompanied by a steady increase in laboratory efforts and husbandry.

A second consideration is that a gene's function is often so essential or pleiotropic that its absence induces early embryonic lethality and in this case the relevance of the gene for a phenotypic trait of interest may be hard to ascertain. Such cases are complicated to address; solutions include inducing mutagenesis at low rates to study mosaic animals, and cell lineage-specific knock-out techniques, which are not yet reported in cichlids.

Rather than loss-of-function, the informative outcome may instead be a more subtle modification: introducing an alternative allele, over-expression or determining the tissue-specific expression pattern of a specific gene or cis-regulatory element. In such cases a knock-in strategy is required. To knock-in using CRISPR, HDR must be used to perform nucleotide replacement or genomic insertion. An established alternative is Tol2 transgenesis (see box 1). Details for experimental design for CRISPR/Cas9 approaches can be found in the next section. In conclusion, it is crucial to carefully formulate the biological question to tackle, choose the right tools, and plan how to infer the phenotypic or molecular effect of your gene edit.

Box 1. **Alternatives to CRISPR/Cas9 genome editing.**

Transcription activator-like effector nucleases (TALENs) are an alternative approach to CRISPR/Cas9 for targeted genome editing. TALENs are composed of a FokI nuclease domain and transcription effector-like DNA-binding domains, which provide target specificity [72]. However, constructing TALENs can be challenging and time-consuming [73]. In cichlids, TALEN genome editing has been applied to Nile tilapia [40,50].

Tol2 transgenesis is an alternative approach to CRISPR/Cas9 for knocking-in sequences. Tol2 transgenesis is based on microinjection in embryos of a mRNA encoding for Tol2 transposase and a plasmid with the transgene of interest, flanked by Tol2 inverted repeats. This leads to random Tol2 integration in the genome, as opposed to precise knock-in with CRISPR-HDR. When employing Tol2 transgenesis, the researcher must decide whether to include a trackable marker (cmlc2:EGFP or similar) and which promoter to use. Also, if the genomic location and number of insertions are relevant, lines with Tol2 insertions may require an extra experimental step to determine the location and number of the insertion(s). If the intent is to perturb the function of a gene by inserting or deleting specific sequences, CRISPR/Cas9 approaches are the more precise option.

Tol2 transgenesis has been applied to tilapia, East African cichlid *A. burtoni* and the Mesoamerican cichlids *Amphilophus citrinellus* and *Archocentrus nigrofasciatus* [16,19,36,56–58] (table 1). The Tol2kit, which allows multisite gateway recombination-based cloning, and contains a trackable heart marker was shown to be effective for tilapia [36,74]. Successful injections have also been reported by presentation at the 2022 Cichlid Genome Editing workshop in *P. demasoni*, using transposase to integrate eGFP into the genome (M.K. 2022, personal communication).

### 2.2. Workflow

**Figure 2.**
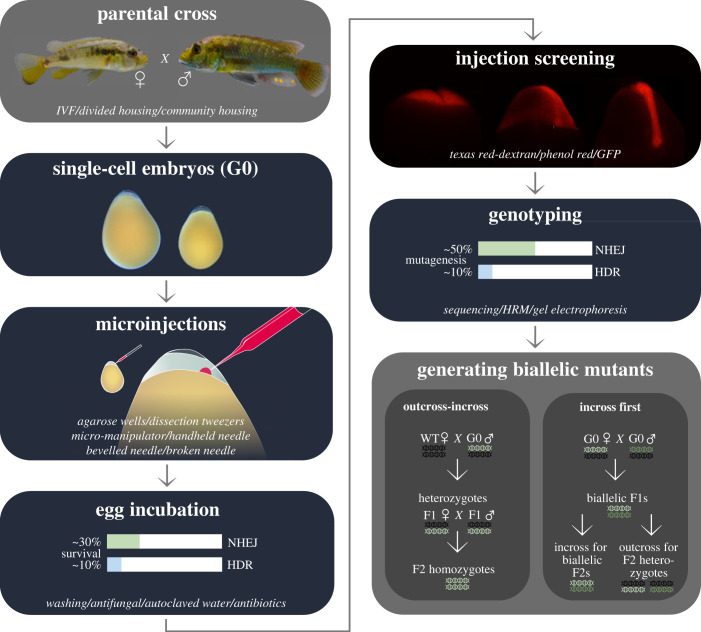
Generalized workflow for genome editing in cichlids. Words in italics indicate alternative options for each stage. Images in ‘single-cell embryos’ and ‘injection screening’ are adapted from Marconi *et al*. [75]. Note that rates of survival (indicating toxicity) and mutagenesis (indicating efficiency) in ‘egg incubation’ and ‘genotyping’ are only from a small number of reports [20,22,41,45]; future optimization will likely change this. In ‘generating biallelic mutants', black DNA symbols indicate WT alleles and green symbols indicate mutated alleles. Different shades of green symbolize different mutations. For simplicity, the genotype of only one cell per individual is represented, but in actuality only some cells in each G0 individual will contain mutations and different cells within the same G0 individual have different mutations.

A generalized genome-editing workflow is applicable across different cichlid species (figure 2). In brief, single cell embryos are obtained by collecting eggs immediately after fertilization, and then injected with a mix containing: injection marker dyes like Texas Red-conjugated dextran or phenol red, and sgRNAs (or crRNAs and tracrRNA) co-injected with Cas9 protein or Cas9 mRNA to induce mutations in the target site. For simplicity, here we refer only to sgRNAs as most genome editing in cichlids has been performed using sgRNAs and the same workflow is applicable regardless of guide system, but see the supplementary information of Li *et al*. [22] for a comparative discussion. The same workflow is broadly applicable to Tol2 transgenesis (box 1), using different injection mix reagents (see above). Embryos can be screened for injection success from early stages by visually screening for the presence or absence of dyes in the embryo cells; however, screening for successful mutagenesis requires collecting tissue to genotype the sequence of interest. Injected animals are a mosaic of WT cells and cells with various mutations. Once identified by genotyping, mosaic mutant animals can be selected for breeding; various incrossing and outcrossing strategies can generate homozygous mutants for phenotypic analysis.

Detailed CRISPR/Cas9 protocols are published for the haplochromine cichlids *Astatotilapia burtoni* [22] and *Astatotilapia calliptera* [20], and Nile tilapia *Oreochromis niloticus* [76]. Also, we now provide a comprehensive, compiled protocol and additional resources intended to be applicable to a large number of cichlids (available here: https://www.protocols.io/view/cichlid-genome-modification-cj5wuq7e and forked versions therein). Consulting these protocols is the primary recommendation for establishing cichlid genome editing in a new setting or species. In the following sections, we assess which stages of the genome editing workflow are most challenging to establish, highlight stages affected by variation in species-specific biology, and identify stages where there is a range of viable technical approaches. Furthermore, until now CRISPR/Cas9 editing in East African cichlids and tilapia has largely been conducted and considered separately, so we include relevant points of comparison to demonstrate the differing advantages and complementarity of the two systems.

### 2.3. Cas9 choice and sgRNA design

Following strategic design, the next stage in CRISPR/Cas9 genome editing is choosing and acquiring reagents and designing sgRNAs. A wide range of Cas9 protein or mRNA options are available commercially. A variety of purchased and homemade Cas9 proteins have been used with similar results in cichlids, though changing the batch of Cas9 used is worth considering when troubleshooting unsuccessful injections. For sgRNAs, commercial chemically synthesized guides have greater quality control than those transcribed *in vitro*, meaning a lower likelihood of other sequences being present in the injection mix that increase toxicity and lower efficiency.

There are multiple software tools available for designing CRISPR sgRNAs. These tools identify suitable target sequences and predict guide efficiency and specificity, which varies between sites. Comparisons of commonly used online tools can be found in [77,78]. A popular tool for genome editing in cichlids is CHOPCHOP (https://chopchop.cbu.uib.no) [79], which is straightforward to use with an interactive display, and users can submit their own reference genome if their species of interest is not already represented in the CHOPCHOP database. For species without a sequenced genome available, it is advised to design guides using the most closely related available species, noting that the off-target calculations will be less reliable. PCR amplification of your region of interest in your species followed by Sanger sequencing is recommended to determine sequence similarity with the related available genome and to ensure the sgRNA sequence and PAM match the prediction.

For loss-of-function mutations, knock-out approaches typically rely on NHEJ introducing errors to the sequence. The aim is to cause a frameshift mutation that introduces premature stop codons, or substantially modifies protein sequence, and so it is advisable to employ guides targeting sites important for protein activity or near the start of the coding sequence of the gene (figure 1; also see supplementary file of Li *et al*. [22] for detailed advice). There are important considerations to take into account when employing NHEJ knock-out approaches, which may render further validation of edited alleles necessary. Creating premature stop codons may lead to mRNA degradation by the nonsense-mediated mRNA decay pathway. Thus, in some cases, it may be useful to verify the influence of particular edits on gene expression. Other aspects to take into account include the possibility of alternative ATG usage and alternative splicing. Gene knock-outs by CRISPR/Cas9 via NHEJ have been reported in Nile tilapia and the East African cichlids *A. burtoni, Pundamilia nyererei,* and *A. calliptera* (table 1).

Other mutations, such as large deletions and allelic exchange, use HDR (figure 1). A template oligonucleotide, which can be prepared by PCR or purchased, containing the desired mutant sequence is presented in addition to Cas9 and sgRNA(s). Due to toxicity, longer single-stranded (ss) DNA templates have a greater effect on survival rates; 100 bp is a suggested upper limit for ssDNA templates. Using HDR, large deletions of non-coding sequences have been generated in tilapia [45] and *A. calliptera* [20] (table 1). No allelic exchange or site-directed transgenesis edits have yet been reported in the literature. HDR is less efficient than NHEJ, resulting in a lower frequency of mutants, G_0_ animals with lower levels of mosaicism, and accordingly lower rates of heterozygotes among outcross F_1_ offspring [20].

It is recommended to sequence the target region in laboratory stocks to identify SNPs before designing CRISPR guides. Natural sequence variation can decrease guide efficiency; therefore avoid selecting guides with SNPs within the target sequence. Multiplexing guides increases the chance of at least one guide causing mutations at a high rate in the injected animals. For example, in targeting *oca2* in the haplochromine cichlid *A. calliptera,* one of two guides employed in multiplex did not result in mutations, despite software predictions of high efficiency [20]. Moreover, cleavage at one guide target site can facilitate cleavage at the site of a nearby, lower-efficiency guide possibly due to chromatin relaxation driven by the DSB generated by the highly active sgRNA [26]. Further, if multiple guides are used targeting sequences within 50 bp of each other, this creates the possibility of generating a deletion between the two sites, increasing the likelihood that the mutation will be disruptive to protein function [26,80]. Although working with sgRNAs in multiplex increases the likelihood of off-target effects, these can be addressed later through outcrossing and backcrossing in breeding schemes.

Mosaicism level is a good indicator for selecting potential broods from the G_0_ population. Nevertheless, the allelic sequence and inheritance rate from mosaic animals to outcross F_1_ offspring can vary between types of mutations. For example, Nile tilapia targeted for *slca45* knock-out showed higher allelic variation in the germline and differential allele heredity: a single-site indel shows the highest inheritance frequency, while a large deletion between multiplexed guides showed the lowest frequency. This may be due to microhomology at one site, low activity at the adjacent site, or their combination [26].

### 2.4. Species

Different species may be used for genome editing depending on the specific research question and feasibility of genome editing. Below, we focus on mouthbrooding East African cichlids and tilapias, where most cichlid genome editing work has been conducted. It is worth noting that most cichlid lines currently grown in a laboratory setting amenable to genome editing are not isogenic. However, there are inbred lines of East African cichlids that have been grown in captivity for decades [3]. The general lack of inbred cichlid lines hampers the definition of ‘WT’ reference strains. It is recommended that researchers perform genome editing in their most inbred lines and keep a careful record for each line of the number of generations spent in captivity.

Between species, the key differences that affect viability for genome editing are the amenability to single-cell embryo collection and the number of eggs per clutch. Obtaining single-cell cichlid embryos requires either control of breeding by timed crosses, close monitoring of natural breeding behaviour, or *in vitro* fertilization (IVF), which offer different levels of convenience and control over timing of fertilization [3]. IVF provides the most control over the timing of fertilization but is the most limited among cichlid species, as it was only performed successfully in tilapia [76] and the Midas cichlid *Amphilophus citrinellus* [58], due to the easily identified pre-spawning characteristics such as protruding genital papilla and spawning-related behaviours, as well as hardiness of the fish and large number of eggs allowing zygote selection. Additionally, in other species abdominal stripping often results in inviable or damaged eggs. Despite attempts to apply IVF to *A. burtoni*, it has not yet been possible to establish it in this species. In the absence of IVF, fertilized eggs can only be obtained after breeding. *A. burtoni* and *A. calliptera* readily breed under timed cross conditions, where females are co-housed with a dominant male separated by a transparent divider, which is removed each morning for short periods while fish are monitored for breeding behaviour [3,14,20,22]. In *A. calliptera*, breeding usually occurs within the hour after divider removal if females are gravid, while *A. burtoni* are reported to typically breed 1–3.5 h after opening the crosses. However, for many species, timed crosses are disruptive and breeding behaviour is very rarely observed when the dividers are removed: species tested include *Rhamphochromis* sp*. ‘chilingali*’, *Tropheops* sp*. ‘mauve*’, *Maylandia zebra, Pseudotropheus demasoni* and *Pseudotropheus saulosi*. For such species, single-cell embryos can be obtained by closely monitoring natural breeding behaviour in community housing. Often clutches are laid within the first hour of facility lights turning on and after water changes. For mouthbrooding cichlids, eggs can be removed by gently spraying water into the female's buccal cavity when breeding behaviour is complete [3]. For egg collection in substrate-brooding species, it may be useful to consult collection methods in other substrate-brooding fishes such as anemonefish [81–83].

Another key difference between species that may affect editing feasibility is clutch size. The number of eggs typically laid per clutch varies widely between species, with a significant impact on ability to troubleshoot injections. In Nile tilapia, 800–1000 eggs can be obtained from a female, which, together with IVF amenability, has greatly facilitated genome editing in this species, resulting in the large number of genes targeted for knock-out analysis (table 1). The average numbers of eggs in East African cichlid clutches are an order of magnitude smaller: 60 in *A. burtoni*, 40 in *A. calliptera,* 50 in *R.* sp*. ‘chilingali’,* 30 in *M. zebra,* 20 in *T.* sp*. ‘mauve’*, 25 in *P. saulosi* and 20 in *P. demasoni*. Within species, clutch sizes vary with individual female size [84]. Importantly, survival rates after injection are typically 20–30% [20,76], so small clutches would result in only a handful of survivors. These species differences in egg number are exacerbated by frequency of breeding: while *A. burtoni* female reproductive cycle is about 21 days, other species reportedly breed at lower frequencies. Longer reproductive cycles can be mitigated by stocking a greater number of females. Ovaprim, a commercially available hormonal spawning inducer, can shorten reproductive cycles and increase clutch size [22]. Despite such interventions to mitigate low egg availability, small clutch sizes can significantly hamper obtaining suitable numbers of mosaic mutants for characterizing and breeding. This is especially so for HDR-mediated edits. With HDR-mediated mutagenesis rates of approximately 10%, and a approximately 10% survival rate following HDR injections, from a clutch of 60 *A. burtoni* embryos only 1 surviving mosaic knock-in animal is expected from two injected clutches. By contrast, targeting the same site for repair by NHEJ in *A. burtoni* with 30% survival rate and 50% mutagenesis rate, a clutch of 60 *A. burtoni* embryos could result in 9 surviving mosaic knock-out animals from a single clutch. Even for species with clutch sizes below 10, injections for NHEJ edits would be expected to yield surviving knock-out mosaics per clutch. Therefore, breeding biology ultimately determines whether HDR-dependent editing is a viable strategy in each species.

### 2.5. Getting started with injections

Micro-injections are the most challenging stage of the workflow to set up for any new non-model species, as obtaining single-cell embryos can be inconsistent and infrequent, and micro-injection techniques can be difficult to optimize [82,85]. When troubleshooting embryo collection and micro-injection for a new species, it can be useful to consult techniques from a range of non-model organisms; biological characteristics such as embryo geometry and size, yolk and membrane material properties, and developmental rate can affect technique suitability. In cichlids, unlike zebrafish crosses, timed crosses do not always yield embryos so multiple attempts across different days are often required, and natural breeding behaviour typically yields embryos every few days. Obtaining embryos therefore presents a time-management problem, where significant proportions of the day (e.g. whole morning) must be set aside for possible injections. It is recommended to dedicate several months to injections, as this maximizes availability to inject when a clutch is laid, and is more productive in the long term. Learning to inject requires hands-on experience so prior injection experience is an advantage; practising with readily available embryos such as zebrafish maximizes the chances of success with precious cichlid clutches. However, an important difference from micro-injections in other fishes is the site of injection: the cells of the embryo are directly targeted (figure 2). It is recommended to avoid rupture of the yolk during injections, as this will negatively affect survival.

The timing of injections is important. The fewer the cell divisions that have occurred at the time of injection, the greater the proportion of cells in injected mosaic animals that will be mutant. Injecting at the 1–2 cell stage is ideal. Injecting at the 4-cell stage is possible; in fact, if the mutation is, or is expected to be, lethal, a lower rate of mosaicism from 4-cell stage injections may be viable when higher rates of mosaicism are not. Furthermore, in fishes the outer membrane surrounding the embryo and yolk, the chorion, begins to harden after fertilization [86,87]. In cichlids this has not been reported as causing an issue for injections, but in other teleosts this chorion hardening can make it difficult for the needle to penetrate [82]. A range of approaches have been employed to manage injection timing, including slowing embryo development and egg-stabilization strategies to hasten injections. Embryo development can be slowed down to allow time to prepare for injections by reducing the ambient temperature. In cichlids, this entails 18–20°C tank water instead of 25–28°C.

To proceed with injections, it is recommended to hold eggs stably in place and to avoid damaging or rupturing the yolk. Egg-stabilization methods that are quick to set up and minimally damaging to the eggs are preferred. Securing cichlid eggs in 2% low-melting agarose wells for injections can increase the speed of injections by twofold [22] compared to securing between the edge of a glass-walled slide chamber and a notched coverslip [57]. Agarose wells are suitable for cichlid eggs due to the large yolk mass, which can be held within the well, leaving the narrow end of the egg facing up with the embryo accessible to the needle (figure 2). The percentage of low-melting agarose used affects the pressure experienced by the egg—higher pressure provides a more secure hold and makes the chorion tense for ease of piercing (see below) but may reduce survival. Custom three-dimensional-printed plastic moulds for agarose wells have been developed with variable well sizes to accommodate minor variation in intra-clutch egg size (https://www.protocols.io/view/cichlid-genome-modification-cj5wuq7e). Moreover, egg size can vary greatly between species and between populations intra-specifically [75,84]. To accommodate such egg-size variation, the correct well size can be determined by testing a clutch with a mould featuring a large range of well sizes, then the parameters can be adjusted in the mould file accordingly to create a custom mould for each species. An alternative approach is to stabilize each egg with blunt dissection tweezers, which removes the need to optimize well size and abolishes the agarose preparation time, but requires greater steadiness of hand. In general, preparation time can be minimized by choosing when to start preparing injection equipment strategically in relation to breeding behaviour. In most cichlid species, this entails starting to prepare agarose wells and injection mixes when egg-laying is first observed, returning to collect eggs after sufficient time is allowed for completion of breeding behaviour (approx. 40 min) to ensure eggs are fertilized. For other organisms, developing a suitable strategy for preparation timing relies on familiarity with the typical course and duration of breeding behaviour.

The concentrations of reagents differ in reports of CRISPR/Cas9 editing between laboratories. Injection mixes contain Cas9 at 80–500 ng µl^−1^ final concentration, sgRNAs at 10–600 ng µl^−1^ combined final concentration, and a dye such as Texas Red-conjugated dextran or phenol red [14,20,22,26,76]. If HDR is employed, the mix should also contain a template for HDR such as ssDNA at 20–100 ng µl^−1^ concentrations. Higher concentrations of Cas9, sgRNAs and ssDNA each improve editing efficiency and consequently mosaicism rates [41], but decrease embryo survival, especially ssDNA templates due to the toxicity of long DNA sequences. Higher concentrations also increase the likelihood of off-target effects, though this can be addressed at later stages through breeding schemes. For more detailed discussion, see the electronic supplementary material of Li *et al*. [22].

Suitable equipment for micro-injection includes MPPI-3 with back pressure unit (ASI), Femtojet (Eppendorf), PV 830 Pneumatic Picopump (WPI) and IM-300 (Narishige) [20,22,26,58,76], all of which require a source of pressurized air, except the Femtojet. A micromanipulator is commonly used to operate the needle positioning, but injections can be performed entirely by hand—this is an operator-specific choice that mainly depends on steadiness of hand and type of previous experience. Suitable micromanipulators include MM33 micromanipulator (ASI) and M-152 micromanipulator (Narishige) [20,22]. The aim is to pierce the chorion and the cell to inject a small volume of injection mix directly into the cell, while avoiding puncturing the yolk, which decreases survival of cichlid embryos [22]. Puncturing the yolk may not be problematic in all other teleosts, as injections of reporter RNA into the yolk in the false clownfish *Amphiprion ocellaris* resulted in surviving fluorescent embryos like injections into the single-cell embryo, albeit with lower ratios of fluorescent embryos and lower levels of fluorescence [83]. Therefore, for establishing micro-injections in other non-model teleosts, it is advantageous to first test alternative injection sites within the egg, as injecting the yolk is easier but could introduce survival issues as in cichlids.

Needle shape and egg handling affect the speed and ease of injecting into the cell without otherwise damaging the egg. Needles for cichlid egg injections are typically shorter than those used for zebrafish. Optimization by trial-and-error is required for the locally available needle puller equipment to yield needles with tips that are thin enough to easily pierce the chorion but still sturdy, as settings to pull needles from glass capillaries vary between needle pullers. Close-up images of suitable needles are shown in Li *et al*. [22]. Distinct cichlid laboratories have different preferred strategies regarding the desired outcome when breaking open the tip of the needle. Breaking the needle with tweezers or against a tissue gives variable outcomes in terms of bore size, angle and roughness, while breaking by beveller, a machine which grinds the tip of the needle at a slanted angle, gives smoother edges and more reproducible bore size and angles. There are conflicting anecdotal experiences on whether a rough edge or clean polished break was most effective for ease of piercing the chorion—which is important for minimizing the force required and reducing the likelihood of puncturing the yolk beneath. In *A. burtoni*, needle bores smaller than 12.5 µm permit maximal survival [22], in agreement with anecdotal data for other species that favour smaller breaks. A suitable injection volume is enough injection mix to cover approximately 1/6 of the base of the cell, which typically requires 1–4 injection pulses depending on the width of the needle bore and the injection equipment.

Injected embryos are cultured individually in plates to facilitate quantifying survival, using tank water, gently shaking at 25–28°C. At this stage, control of infection is important. Different combinations of approaches have been taken across different laboratories: autoclaving water, adding anti-fungal methylene blue (1 mg l^−1^), adding antibiotics penicillin and streptomycin (Sigma P4333 with 10 000 units penicillin and streptomycin 10 mg ml^−1^; diluted 1 : 1000), and washing eggs before injection in diluted bleach. One consideration to take into account is that researchers repeatedly exposed to anti-fungals and antibiotics can develop contact dermatitis (allergies) to these substances. An effective combination of approaches for the available water quality may have to be defined by trial-and-error. What is agreed upon is that daily water changes are required at least until embryos are hatched from the chorion, to avoid infection.

### 2.6. Screening and genotyping

An initial screen for introduction of injection mix into developing embryos determines if an injection has been successful. Incorporation of the injection mix into dividing cells can be monitored through co-injection of Texas Red-conjugated dextran, phenol red, or GFP (expressed from injected *gfp* mRNA or Tol2 plasmids/insertions). An absence of fluorescence or dye presence in the embryo indicates failure to pierce the cell or to deliver the injection mix. The yolk of injected cichlid embryos is strongly autofluorescent if the injected embryo has died.

Screening for genome edits requires genotyping, for which there are a range of methods that vary in effort, cost, and informativeness [80]. These include Sanger sequencing [20], next-generation sequencing [22,26], high-resolution melt (HRM) analysis [26], a fluorescent PCR approach called CRISPR-STAT [22,88], and gel electrophoresis to separate PCR product sizes after restriction enzyme digests or if a deletion has been generated (by HDR or two closely spaced guides) [20]. We recommend consultation of the supplementary material of Li *et al*. [22], where a detailed and comprehensive comparison of relevant genotyping approaches is provided. Sequencing is the most informative as it enables characterizing the mutations. Mosaic and heterozygous mutants can be readily identified by a drop in sequencing trace quality at the sgRNA cut site, indicating that multiple different sequences continue from that point. In heterozygotes, 50% of the sequences will be the WT allele and 50% the mutant allele. In a mosaic mutant, there may be many different mutant sequences from different cells contributing to the genotyped sample. In both heterozygotes and mosaics, multiple sequences can be resolved from directly Sanger sequencing a single PCR product using chromatogram dephasing software [89], bypassing the need for cloning. The free online software ICE by Synthego (https://ice.synthego.com) is recommended for chromatogram dephasing. Synthego ICE takes as inputs the sgRNA sequences, and sequencing files (.ab1 format) for test samples and a WT sample. For each test sequencing file, the output is a list of the sequences contributing to the sequencing trace, with an estimated proportional contribution from each sequence. For mosaics, these proportions can estimate the proportion of cells within each animal that carry WT or mutated alleles. For heterozygotes, approximately 50% of contributing sequences are WT, and the highest-proportion mutant sequence can be taken as the mutant allele sequence. Different genotyping methods can be employed when most suitable for the workflow requirements, such as sequencing for characterizing mutations followed by HRM for population screens.

All genotyping methods require extracting DNA, which necessitates either sacrificing the embryos or raising the fish to adult stages large enough to take tissue samples by fin clipping or skin swabbing. For this reason, targeting genes with a clear knock-out phenotype appearing early in development greatly facilitates protocol optimization across different species. Genes involved in the melanin synthesis pathway are ideal for this purpose, as they are often non-essential for survival and large numbers of melanin-bearing melanophores are visible on the yolk sac 4–6 days post-fertilization (dpf) in East African cichlid species [20,90,91] and 3 dpf in Nile tilapia [29]. Targeting melanin synthesis genes (table 1) leads to reductions in numbers of visible melanophores at these stages, enabling straightforward and rapid assessment of CRISPR/Cas9 editing success without need for genotyping every clutch. It is therefore recommended to initially optimize protocols by targeting one of these genes before targeting other genes of interest. This has proven a useful approach in cichlids [20,22,26,28] as with other model and non-model teleost organisms [81,85,92–94]. In *A. burtoni,* knocking out *tyrosinase* has the additional effect of generating autofluorescent cells, presumably due to accumulation of a fluorescent metabolite derived from tyrosine [22], adding a further visual confirmation of genome editing success.

### 2.7. Breeding mutant lines

Following the generation of G_0_ mosaic animals, the aim is typically to generate biallelic mutants for phenotypic analysis. Here, we use the general term biallelic to describe all cases where both alleles are mutant (regardless of whether mutations are identical or different), to distinguish this from the more specific term homozygous when both alleles have identical mutations [95]. An exception to the aim of generating biallelic mutants is when mutations are expected to be lethal, as only animals with low rates of mosaicism are viable in such cases. For lethal mutations, phenotypic analysis can be carried out in the mosaic generation and where haploinsufficiency is expected also in heterozygous F_1_ offspring. Similarly, in slow-maturing species it may only be practical to analyse phenotypes in the mosaic generation; in this case highly mutant mosaics are desirable.

A prerequisite for breeding biallelic mutants is germline transmission of the induced mutations from the G_0_ animals to the F_1_ generation. Germline transmission typically reflects rates of mosaicism [20,76] and so can be inferred for each G_0_ individual from its proportion of mutant cells in somatic tissues (e.g. tail fin). Alternatively, germline transmission can be assessed in males by collecting sperm for DNA extraction and genotyping, an approach used in tilapia [76], which could be applied to other cichlids by establishing sperm collection. It is advisable to select animals with high germline transmission rates as founder individuals for breeding mutant lines.

Breeding schemes to generate biallelic mutant lines vary according to priorities. Ideally, a biallelic mutant line would be homozygous for a single mutation. This requires outcrossing mosaic animals with WT animals and genotyping F_1_ offspring to identify heterozygotes with identical mutations (figure 2). In cichlids, outcrossing is most efficiently achieved by housing a single mosaic male with multiple females. In tilapia, genetically all-male G_0_ injection clutches can be obtained via a combination of hormonal sex reversal and crosses [76]. This requires additional generations of breeding prior to injections; where this is not possible, males can instead be selected from the mixed G_0_ generation.

Genotyping F_1_s by sequencing is advantageous as it enables specific selection of the desired mutations. For knock-out approaches where loss of gene function is the aim, indels that cause frameshift mutations and premature stop codons near the start of the coding sequence are typically preferred. Moreover, sequencing enables validation that the intended mutation has occurred. For example, CRISPR/Cas9 editing with a HDR template can result in additional random insertions of template-derived sequences (including truncated or tandem insertions at the targeted site, or other off-target insertions in other loci), as with other genome editing techniques that include a template donor sequence [20,80,96]. Unintended mutations at the targeted site may be distinguished by sequence analysis or gel electrophoresis (as they will lead to a PCR product of different length, if the genotyping primer-binding sites were not affected by the insertions). Other unintended structural variation can occur with HDR, such as tandem duplications that cannot be identified by routine PCR, instead requiring qPCR-based genotyping, targeted locus amplification sequencing or nanopore sequencing [97]. Heterozygotes with desired and matching mutations can be incrossed to generate offspring in Mendelian ratios, including one quarter homozygotes [14,22]. The one quarter of the offspring that are WT serve as an essential control for genetic background when analysing phenotypic effects of the mutations, as can the half of offspring that are heterozygotes. Mutations in the analysed generation can be validated by sequencing, and any difficulties in genotyping by routine PCR (such as unexpected offspring ratios) can be investigated by additional sequencing methods [97]. As further control, multiple independent lines should be analysed where possible. This is because homozygotes derived from the same founder may carry particular genetic backgrounds that confound the analysis. Secondly, it is possible that different induced mutations may not have the same loss-of-function phenotype (for example, when some are hypomorphic). Moreover, having multiple lines reduces the risk of off-target effects influencing the analysis. By generating multiple independent homozygous mutant lines, and either analysing them separately or crossing lines together to generate biallelic mutants, it can be confirmed that the mutations have similar effects.

In some cases parental effects can confound analysis in the next generation, such as *dicer* mutants in zebrafish not being lethal as expected due to maternally deposited Dicer protein [98]. For cichlids or other non-model organisms with long generation times, it may well be infeasible to wait an additional generation to fully characterize phenotypes with such effects.

A multi-generation approach with outcrossing first best controls for off-target effects and similarly confounding genetic factors. However, there are two drawbacks to the outcross-then-incross approach. Firstly, identical mutations may only be found in a low proportion of heterozygous offspring, yet high numbers of breeding heterozygotes facilitate obtaining desired numbers of F_2_ offspring, as well as managing aggression within the F_1_ breeding stock. Resolving this trade-off in species with smaller clutch sizes would require obtaining and genotyping many clutches of F_1_ offspring. A more pragmatic solution is to cross any heterozygotes with desired mutations regardless of whether the mutations are identical to each other. Moreover, non-model teleosts are typically slower maturing than model organisms. Among cichlids, some species take several months to reach sexual maturity. The generation times of species with published CRISPR/Cas9 editing are approximately 6 months for Nile tilapia, approximately 4 months for *A. burtoni* [22]*,* approximately 8 months for *A. calliptera*. Longer generation times cause a multiple-generation breeding scheme to be inconvenient, likely requiring a line to be maintained in the laboratory and later analysed by a different researcher. An alternative approach is to analyse earlier generations to reach an answer more quickly: this can be achieved by incrossing mosaic founders (G_0_) to create biallelic mutant animals in the F_1_ generation (figure 2). Incrossing the resulting F_1_s generates biallelic F_2_s, while outcrossing biallelic mutants results in 100% heterozygous offspring (figure 2), which can be used to generate mixed WT and mutant clutches for phenotype comparisons as described above. In *A. calliptera* targeting the melanin synthesis gene *oca2*, this approach successfully resulted in mutant animals with an amelanistic phenotype within a single generation, and similarly resulted in biallelic mutants for HDR-mediated mutagenesis [20]. However, immediately incrossing G_0_ animals in this way does not control for off-target effects. To address this, an improved strategy is to derive lines by incrossing lines from independent founders, ideally generated by two independent sets of sgRNAs. This approach relies on the assumption that the differing off-target affinities of the different guides cause off-target mutations to happen at such low frequency that they are unlikely to occur in two different founders. Given the long generation time in cichlids, this may be the most feasible strategy with adequate off-target effect control. That said, every outcross reduces the potential for off-target effects (note that genetically linked off-target effects are only eliminated by recombination events and therefore usually require more outcrosses), thus outcrosses should be factored in where possible.

## 3. Possible avenues for improvement of the state-of-the-art

We argue that implementation of CRISPR/Cas9, particularly with mutagenesis as a goal via NHEJ-mediated repair, is now feasible for most cichlid research laboratories with access to basic molecular biology reagents and equipment. The consensus in the community is that, for most cichlid species other than tilapia, current efficacy rates and clutch sizes are too low to allow for easy use of HDR-based approaches, such as for allelic exchanges. For reporter lines, Tol2 transgenesis (box 1) is currently the viable alternative. For HDR-based CRISPR/Cas9 editing to work with current efficiency rates in such species, it would require maintaining a large number of crossing tanks to obtain and inject clutches frequently. For these reasons, cichlid researchers are, as a community, far from the implementation of more sophisticated genome editing approaches, including conditional knock-outs, lineage tracing, transcriptional modulation (CRISPR interference or CRISPR activation) or epigenome editing. However, the viability of HDR-dependent approaches could quickly change with upcoming techniques that improve HDR efficiency [68]. Adoption in cichlids of CRISPR/Cas9 advances made in model species will be key to this.

Improvements to HDR efficiency include use of different donor templates: in addition to ssDNA, the only HDR template used so far [20,45], possible donors include plasmids, PCR-derived double-stranded DNA (dsDNA), and Cas-guided transposases [68,99–103]. Specific design of donor templates can improve efficiency, namely choice of strand, length of the 3' homology arm, and distance from the DSB to the knock-in site [45,104–106]. The ratio of concentrations of Cas9, guide RNA and donor template can also affect HDR efficiency and toxicity [107]. Furthermore, biotinylated repair donor constructs can prevent concatemerization of injected dsDNA and enhance its binding to Cas9 [68,108,109]. Finally, activators and inhibitors of HDR and NHEJ pathways can be employed to improve HDR repair rates [110].

Further, realistic efforts in the near future to further improve the versatility of CRISPR-based approaches in cichlids could include the use of other RNA-guided nucleases with different PAM sequences, to allow for a more flexible targeting of the genome. A good example is Cas12a (Cpf1), with a preference for a 5'TTTV PAM, which makes AT-rich regions more accessible to editing [111,112]. Such improvements could help bridge the gap between genome editing technologies in model fish species, like zebrafish and medaka, and cichlid fishes. In the long term, single-base editing systems such as CRISPR-X that induce somatic hypermutations or Cas9 nickase-cytidine deaminases fusion systems that cause C to T conversion [93,113–115] are some of the cutting-edge techniques that would further help in expanding the cichlid toolbox of genome editing and increase versatility and precision.

Many published studies concerning editing the genome of cichlid fishes have targeted genes with roles in pigmentation (table 1). Due to the ease of screening for amelanistic phenotypes, targeting these genes with published, efficient sgRNA would be a good start for laboratories establishing their own CRISPR/Cas9 setup. In addition, we propose that pigmentation genes can be targeted as a co-CRISPR to streamline screening. The principle of co-CRISPR is simple: by injecting published and efficient sgRNAs targeting genes responsible for a dominant, visible phenotype along with sgRNAs targeting your gene of interest, one can select for injected embryos with visible phenotypes, where Cas9 was active. This selection according to Cas9 activity leads to an enrichment of your edits of interest. This strategy should be avoided if the biological process under study is affected by loss of pigmentation. If undesired, co-CRISPR mutations can be crossed out in subsequent generations. Alternatively, if your co-CRISPR gene is in close linkage with your gene of interest, the visible phenotype may be used to bypass genotyping in subsequent mating. This approach has been originally implemented in the nematode *C. elegans* [116], and was used in Atlantic salmon by co-targeting the pigmentation gene *Alb* [117].

The development and improvement of a range of satellite technologies would benefit genome editing technologies in cichlids. For example, given the current low efficacy of genome editing due to low brood sizes and mouthbrooding, efforts to obtain single-celled fertilized embryos more regularly or at higher frequency are valuable [22]. Investigating alternative delivery methods such as viral delivery, embryo electroporation, unfertilized oocyte injections that generate G_0_ mutants, or sperm electroporation into oocytes may enable upscaling of cichlid genome editing [118–120]. Development of robust protocols for long-term cryopreservation of sperm will help in stock management and sharing of fish lines between laboratories (shipping frozen gametes instead of fish), and thus represent a good reduction alternative (in relation to the 3R principles of replacement, reduction and refinement). Cryopreservation of gametes is dependent on the development of IVF protocols to resurrect mutant lines. In addition, IVF would also remove dependency on carefully timed crosses. Sperm for IVF can be attained by dissection of euthanized animals, but this is not always desirable as cichlids may be maintained at fairly low numbers per tank. Obtaining gametes for IVF without euthanizing animals is more easily achievable in non-mouthbrooding than mouthbrooding cichlids, due to the elaborate mating behaviour that precedes egg laying in mouthbrooding cichlids.

An increasing number of cichlid laboratories using genome editing translates to an increase in the number of cichlid lines and alleles produced. In order to cite and share particular genetic reagents in a reproducible, transparent and efficient way, we strongly recommend the adoption of nomenclature guidelines. Guidelines currently in place for zebrafish could be easily adopted at a first stance (https://zfin.atlassian.net/wiki/spaces/general/pages/1818394635/ZFIN+Zebrafish+Nomenclature+Conventions). Caveats include the need for a dedicated team of curators/moderators to assist and register gene and allele naming, and to attribute line designations to particular laboratories or institutions (https://zfin.org/action/feature/line-designations). We invite the cichlid research community to discuss these next steps at future community meetings.

Lastly, the development of platforms for resource and protocol sharing will largely benefit the cichlid research community, for example by increasing communication and knowledge transfer, as well as sharing mutant lines and reagents. In this regard, we propose the following measures: (1) enrolment of cichlid research members in a common Slack workspace managed by the community (@cichlidscience2022.slack.com); (2) preparation of a centralized database using online platforms; and (3) sharing protocols, using for example protocol.io (with the following compiled protocol and resources as an example: https://www.protocols.io/view/cichlid-genome-modification-cj5wuq7e). All these measures should be free, open to all, and easily accessible, with Internet connection as the only requirement.

## 4. From the state-of-the-art to new frontiers in cichlid genome editing

**Figure 3.**
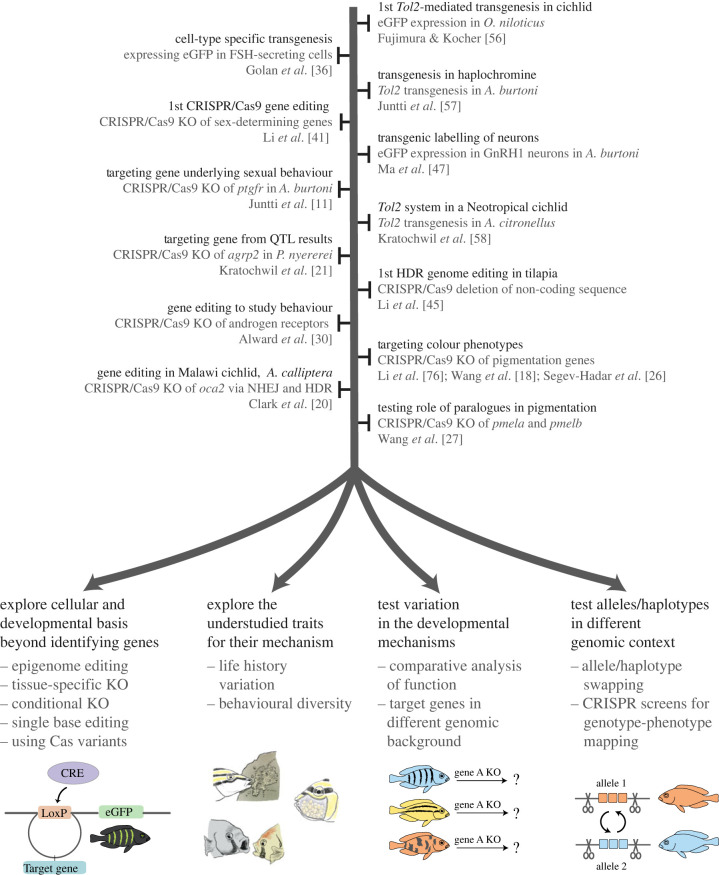
Overview of the state-of-the-art and the emerging future of genome editing in cichlids. The key studies in genome editing of cichlids are shown in a chronological order. The top illustrates the progression of applying CRISPR/Cas9 and Tol2 system in the past decade to study diverse traits such as behaviour and pigmentation in cichlids. The bottom highlights the possible future directions of genome-editing and transgenic techniques to push the frontiers of uncovering evolutionary and developmental basis of phenotypic traits in a more comprehensive manner.

With the availability of transgenic and genome-editing techniques, studying evolution and development of phenotypes in cichlid fishes has become increasingly exciting in the past decade. These techniques provide the opportunity to experimentally test hypotheses regarding the implication of genes and mutations that correlate with phenotypic divergence (figure 3). Moreover, they open up new avenues to not only study genotype–phenotype relationships, but also the cellular and developmental mechanisms that connect them [121,122]. Applied to tilapia, genome editing can develop solutions for aquaculture challenges and investigate the genetic, developmental, and physiological mechanisms underlying commercially relevant traits [123]. Genome editing for aquaculture purposes has been widely reviewed [72,124–126] including from biosafety, sustainability, and welfare perspectives [127–129] so here we focus on studying the development and evolution of phenotypes.

Seeking techniques that are well established in model organisms, such as zebrafish, has been and still is the first logical step to address the genetic and mechanistic basis underlying the evolution of phenotypes in cichlids. Currently, evolutionary–developmental (evo-devo) research on cichlid fishes and non-traditional model organisms more generally tends to take one of two paths. The first path is a more development-focused approach that studies phenotypic traits that cannot be analysed in more traditional model organisms to understand their genetic, developmental and mechanistic basis (figure 3). The second direction is a more evolution-focused approach that follows up on candidate genes from traditional genotype–phenotype mapping studies such as GWAS and QTLs. Here, the focus is on both providing experimental evidence for the causal implication of candidate alleles and genes, as well as studying how these genetic changes have led to developmental changes that drove the phenotypic divergence. Below, we provide examples of how the field is already pushing the frontiers of understanding the evo-devo basis of phenotypic traits and speculate how the adoption of cutting-edge and future techniques might drive the research field forward.

Cichlids offer a wide array of traits that cannot be found in other vertebrates or where studying them is more challenging. This includes, for example, social behaviours [30], spiny-rayed appendages [14], craniofacial and teeth morphologies [130,131], colour pattern diversity [21,132,133], or sex determination [134]. Through cichlid studies we have already obtained exciting insights into the implication of specific genes, such as androgen receptor genes, in determining social status of cichlids, or the BMP*-gremlin-shh* gene network in regulating fin development [14,30]. However, we still lack a thorough understanding of the mechanistic roles of these genes in development. To address this, it could be useful to generate tissue- and cell-specific reporter lines using either transgenic reporter constructs [74] or by knocking-in reporter genes [135] that have been standardized in zebrafish. Adapting such techniques to replace genes underlying diverse phenotypes with reporter genes would permit exploration of the mechanistic role of the gene in development of the trait. For example, for *oca2,* a gene associated with albinism and where a knock-out has already been established in *A. calliptera* [20], *oca2* could be replaced with GFP. This would allow tracing the development of melanophores under different conditions including gene knock-outs or different genetic backgrounds. For other genes that are implicated in complex and less understood processes, for example the role of the gene *pax7a* in the orange-blotch phenotype [136,137], knocking-in GFP would allow identification of its spatio-temporal activity during development. Moreover, it would facilitate studying with more mechanistic detail how regulatory variation of *pax7a* translates to the blotch phenotype. Another example is the gene *agrp2*, required for development of stripes [21]: like *pax7a*, it is unclear when and in which cells regulatory variation of this gene causes the phenotype. As HDR-dependent techniques, generating such reporter lines would require adopting more efficient HDR approaches or using Tol2 transgenesis, as discussed in the previous section. For traits shared between tilapia and other cichlids, it may be beneficial to study these in tilapia, where higher clutch sizes make HDR-dependent techniques more viable. A further ambition for development-focused studies in cichlids would be to explore mechanisms of pleiotropic genes that have a detrimental effect if mutated constitutively. For instance, genes involved in BMP signalling are important for cranio-facial adaptive traits but also govern embryo development [138]. For this, conditional knock-outs generated by Cre- or Gal4/UAS-controlled CRISPR mutagenesis could be a way forward in the long term [139].

While studying the development of cichlid phenotypes, even if comparatively between a few species, the approach typically does not directly address the evolution of the underlying mechanisms. While this can be challenging to study, the diversity of natural phenotypic variation in cichlids is an asset that we must exploit to study the evolution of traits across species. The abundance of species amenable to genome manipulation and the relative feasibility of generating knock-outs make it possible to perform comparative knock-outs of genes with known function. This would allow us to explore the evolution of the mechanisms underlying diverse phenotypes across a variety of genomic backgrounds and test questions regarding the robustness and evolvability of the developmental mechanisms. As knock-outs can be achieved through NHEJ, providing such an evolutionary perspective to developmental mechanism-driven research does not require any substantial advances to CRISPR/Cas9 technology in cichlids.

Turning to the more evolution-focused approach in cichlid evo-devo, currently available CRISPR/Cas9 technology can provide evidence for causal roles of genes identified by evolutionary genetics approaches. Owing to the large sequencing projects [6,9] and pedigree-based mapping panels [21,140–142] that have been conducted in the last decade we now have rich data sets on genotype–phenotype mapping and screens for signatures of selection. As these are correlative, there is a lack of confidence in the causal implication of genes in phenotypes. Moreover, for screens for signatures of selection, the traits affected by the underlying allelic changes often remain unknown. Genome editing allows us to now rigorously test these hypotheses by knocking out candidate genes. Adopting engineered variants of Cas9, other Cas nucleases, and single-base editing systems⁠ can bring flexibility while choosing genome-editing targets, as discussed in the previous section. Cutting-edge technologies will become particularly important when the field moves from knocking out gene function to taking a more precise approach by replacing specific alleles, given improvements in HDR efficiency or obtaining fertilized eggs. Looking ahead, it might even be possible to not only swap single alleles or small sequence stretches but larger sequences. With such technologies it would be in principle possible to test complete haplotype stretches in the genomic background of another species. Once optimized and made feasible, it may even be possible to conduct screens for multiple candidate genes/alleles to map genotypes to phenotypes. In some cases, mapping by genome editing could partly or fully replace long and cumbersome genotype–phenotype mapping methods such as pedigree-based mapping.

In summary, going beyond the state-of-the-art, leveraging advanced techniques to uncover mechanisms and addressing the evolutionary context at the same time will truly take us towards a comprehensive and holistic view of cichlid fish evolutionary–developmental biology that puts an emphasis on both the EVO and the DEVO as well as their integration.

## Data Availability

This article has no additional data.
